# Lumbopelvic Muscle Changes Following Long-Duration Spaceflight

**DOI:** 10.3389/fphys.2019.00627

**Published:** 2019-05-21

**Authors:** Kyle P. McNamara, Katelyn A. Greene, Austin M. Moore, Leon Lenchik, Ashley A. Weaver

**Affiliations:** ^1^ Center of Injury Biomechanics, Virginia Tech - Wake Forest University School of Biomedical Engineering and Sciences, Winston-Salem, NC, United States; ^2^ Department of Radiology, Wake Forest School of Medicine, Winston-Salem, NC, United States

**Keywords:** astronaut, cosmonaut, microgravity, muscle atrophy, muscle attenuation, computed tomography

## Abstract

Long-duration spaceflight has been shown to negatively affect the lumbopelvic muscles of crewmembers. Through analysis of computed tomography scans of crewmembers on 4- to 6-month missions equipped with the interim resistive exercise device, the structural deterioration of the psoas, quadratus lumborum, and paraspinal muscles was assessed. Computed tomography scans of 16 crewmembers were collected before and after long-duration spaceflight. The volume and attenuation of lumbar musculature at the L2 vertebral level were measured. Percent changes in the lumbopelvic muscle volume and attenuation (indicative of myosteatosis, or intermuscular fat infiltration) following spaceflight were calculated. Due to historical studies demonstrating only decreases in the muscles assessed, a one-sample *t* test was performed to determine if these decreases persist in more recent flight conditions. Crewmembers on interim resistive exercise device-equipped missions experienced an average 9.5% (2.0% SE) decrease in volume and 6.0% (1.5% SE) decrease in attenuation in the quadratus lumborum muscles and an average 5.3% (1.0% SE) decrease in volume and 5.3% (1.6% SE) decrease in attenuation in the paraspinal muscles. Crewmembers experienced no significant changes in psoas muscle volume or attenuation. No significant changes in intermuscular adipose tissue volume or attenuation were found in any muscles. Long-duration spaceflight was associated with preservation of psoas muscle volume and attenuation and significant decreases in quadratus lumborum and paraspinal muscle volume and attenuation.

## Introduction

Significant skeletal muscle atrophy has been observed in crewmembers of long-duration spaceflights and persists for months following spaceflight, particularly in the legs and back (LeBlanc et al., 2000; Ploutz-Snyder et al., 2015; Chang et al., 2016). Muscle volume decreases due to prolonged exposure to microgravity during long-duration spaceflight may increase the risk of astronaut injury while on mission, upon return to earth, or later in life (LeBlanc et al., 1998, 2000; Adams et al., 2003; Lang et al., 2004; Ploutz-Snyder et al., 2015). Studies on muscle atrophy during long-duration spaceflight have primarily focused on the lower extremities, but recent spaceflight and bed rest studies have sought to characterize loss in the supporting spinal musculature (Cao et al., 2005; Ploutz-Snyder et al., 2015; Chang et al., 2016; Macias et al., 2017). Historical magnetic resonance imaging data from 17-day missions have shown a 10% volumetric loss in the intrinsic back muscles (rotatores, multifidus, semispinalis, spinalis, longissimus, and iliocostalis) and 5% volumetric loss in the psoas muscles, while 16- to 28-week missions resulted in 16% loss in the intrinsic back muscles and 5% loss in the psoas muscles (LeBlanc et al., 2000). Long-duration bed rest studies have been used as corollary for spaceflight studies of muscle degradation as they are both associated with increased muscle soreness, decreased muscle performance, and decreased postural stability (Nicogossian et al., 1994; LeBlanc et al., 2000; Cao et al., 2005; Muir et al., 2011; Macias et al., 2017). Nevertheless, these studies may not adequately capture the full degree of lumbopelvic muscle degradation that occurs with long-duration spaceflight (LeBlanc et al., 1992). Back muscles are likely used in bed rest to adjust position. In the 1-g environment on Earth, lumbopelvic muscles are activated in a tonic fashion that is in anticipation of dynamic loads with various up-right movements, offering segmental control and stability of the spine (Hodges and Richardson, 1997; Moseley et al., 2002). In spaceflight, some spinal loading is achieved through intravehicular activities such as treadmill and resistive exercise as well as any extravehicular activities while on the mission. However, these activities do not produce the same type and magnitude of gravitational loading experienced on Earth. Moreover, during spaceflight, even with the use of advanced exercise equipment, microgravity causes an absence of anticipatory loads, disrupting the feedforward mechanism that normally activates the lumbopelvic muscles (Winnard et al., 2017).

A chief goal of NASA’s Human Research Roadmap is to investigate whether atrophied and weakened spinal musculature paired with vertebral bone loss will affect their quality of life upon return to Earth, since lost muscle and bone mass are not immediately restored (LeBlanc et al., 2000; McCarthy et al., 2000; Carpenter et al., 2010; Caldwell et al., 2012). In particular, the combination of spaceflight-induced loss of muscle strength, sensorimotor impairment, reduced postural stability, and bone loss may predispose astronauts to herniated discs (Johnston et al., 2010) and vertebral fracture due to dynamic loading encountered in spacecraft launches and landings and/or due to falls in the years following spaceflight (LeBlanc et al., 2000; Lang et al., 2004; Muir et al., 2011; Wood et al., 2011). In addition to risk of injury, in-flight back pain is a known effect of spaceflight with the majority of crewmembers experiencing some degree of back pain during flight (Sayson and Hargens, 2008; Kerstman et al., 2012; Pool-Goudzwaard et al., 2015). The most common location of spaceflight-induced back pain occurs in the lumbar region. It is postulated that both disc and muscle changes are contributing to this phenomenon (Sayson and Hargens, 2008; Belavý et al., 2011; Hides et al., 2016). Spinal musculature provides support for the vertebral column. Chronic back pain studies in terrestrial populations have linked decreases in the paraspinal muscles and psoas to back pain (Hodges and Richardson, 1996; Hides et al., 2011), though the same correlation is not clear with regard to muscle attenuation, a measure of muscle density in which lower values suggest fat infiltration, and back pain (Danneels et al., 2000; Kamaz et al., 2007).

Early exercise equipment in space consisted of bicycle machines, which provided aerobic training that was ineffective in combating muscle loss, highlighting the need for on-board resistance-based exercise equipment (Thornton and Rummel, 1977). Long-duration International Space Station (ISS) missions have retained the important focus on cardiovascular health with the presence of a cycle ergometer with vibration isolation and stabilization (CEVIS) and a treadmill with vibration isolation and stabilization (TVIS) and its 2009 replacement, the second-generation treadmill (T2; Petersen et al., 2016). Efforts to minimize muscle degradation on ISS missions resulted in the introduction of the interim resistive exercise device (iRED), which used elastomers to mimic weight-bearing exercise as a complimentary in-flight countermeasure (Shiba et al., 2015). This was improved upon in 2010 with the introduction of the advanced resistive exercise device (aRED), which simulated inertial loads experienced at earth-based gravitational pull.

Volumetric changes in spinal musculature occurring as a result of prolonged spaceflight can be measured using computed tomography (CT) scans. Additionally, CT scans can provide an insight into the development of intermuscular adipose tissue deposits as well as intramyocellular lipid deposits in the myocyte cytoplasm through measuring muscle attenuation values (Aubrey et al., 2014; Lee et al., 2017). The combination of these analyses allows for investigation into the influence of long-duration spaceflight on muscle health. The objective of this study was to assess pre- to post-flight changes in the psoas, quadratus lumborum, and paraspinal muscles from lumbar spine CT scans of crewmembers (*n* = 16) on 4- to 6-month iRED-equipped missions. It was hypothesized that the lumbopelvic muscles would experience post-flight decreases in muscle volume and attenuation in these iRED-era missions.

## Materials and Methods

Retrospective CT scans were obtained from the Life Sciences Data Archive and Lifetime Surveillance of Astronaut Health project. Written informed consent was obtained from each subject, and the study protocols were approved by the Wake Forest School of Medicine and National Aeronautics and Space Administration Institutional Review Boards. Helical CT images at the level of the L1 and L2 vertebrae were acquired using a GE HiSpeed Advantage at 80 kVp and 140 mA with 3 mm slice thickness and 0.94 mm pixel size (Lang et al., 2004). Scans were obtained for 16 crewmembers made up of astronauts and cosmonauts (average age, 45.9 years; 15 males and 1 female) who flew missions between 4 and 6 months in duration during the period when iRED was available to crewmembers. Pre-flight CT scans were performed 30–60 days before launch, and post-flight CT scans were performed within 7–10 days after landing (Lang et al., 2004).

**Figure 1:**
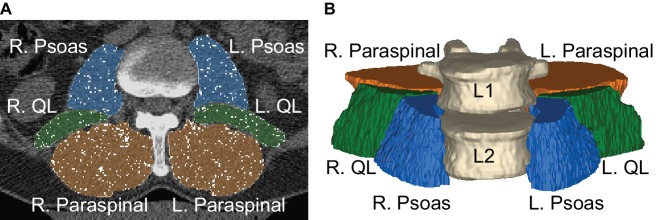
**(A)** Manual muscle segmentations of the psoas (blue), quadratus lumborum (green), and paraspinal muscles (orange) on a CT scan. Fat voxels (white) have been removed from each of the muscle masks. **(B)** Three-dimensional view of the muscle segmentations represents muscle volumes that span the L2 vertebral body.

The psoas, quadratus lumborum, and paraspinal (consisting of the erector spinae and multifidus) muscle groups were segmented and analyzed from the CT images to characterize lumbopelvic muscle volume changes (Figure 1). Segmentation was standardized using the morphology of the L2 vertebral body to ensure consistent regional analysis on a per-crewmember level. Segmentation was performed in the axial view starting with the slice superior to the L2 vertebral body and ending on the slice inferior to the L2 vertebral body. The pre- and post-flight scans per crewmember were quality checked to make sure they encompassed the same regions and had masks of the same height. Semi-automated segmentation was conducted using Mimics software (v20, Materialise, Leuven, Belgium). An initial Hounsfield Unit (HU) threshold for muscle (−29 to 150 HU) was applied and the resulting mask was manually refined to ensure voxels within the external boundary of each lumbopelvic muscle were included in the mask (termed “total muscle mask”). Once all lumbopelvic muscles were segmented, the axial slices were checked for consistent muscle boundary definitions on an intra-subject level. Once lumbopelvic muscle masks were validated, a fat threshold was applied (−190 to −30 HU; Prado et al., 2009) to define voxels representing intermuscular adipose tissue (IMAT; termed “fat mask”) within the “total muscle mask.” A Boolean operation was then performed to subtract the “fat mask” from the “total muscle mask” to obtain the “true muscle mask.” Any fat voxels on the outer layer of the muscle mask were determined to be muscle fascia and were manually removed from the muscle mask to create a “fat infiltration mask” representing IMAT.

From these segmentations, pre- to post-flight changes in muscle volume were quantified. The volume of each true muscle mask was calculated by determining the volume of a 0.94 mm × 0.94 mm × 3.00 mm voxel and multiplying by the number of voxels in the muscle segmentation. Similarly, the volume of IMAT was determined using the same method. The gross fat percentage for each muscle was calculated as the percentage of the combined true muscle and fat infiltration masks occupied by the fat infiltration mask. Muscle and fat attenuation (measure of lipid concentrations in the masks) were calculated as the average HU for each mask. The pre- to post-flight percentage change for the volume and attenuation of each mask was calculated on a per-crewmember level.

Statistical analysis was performed using JMP Pro (JMP^®^, Version 13. SAS Institute Inc., Cary, NC, USA). Shapiro-Wilk tests were performed on all muscle changes to evaluate for normally distributed results. Paired *t* tests (*α* = 0.05) were performed to determine the mean change in lumbopelvic muscle and fat volumes on a per-crewmember level. Paired *t* tests (*α* = 0.05) were also performed to determine the mean change in muscle and fat attenuation on a per-crewmember level. Finally, paired *t* tests (*α* = 0.05) were performed on the percent change in the volume and attenuation of muscle and fat in the pre- versus post-flight scans. All results are presented as means and standard errors.

A subset consisting of six crewmembers had in-flight workout logs of time spent using CEVIS and TVIS as well as frequency of iRED usage. The crewmembers also had pre- and post-functional fitness assessments consisting of peak torque on 60° knee and trunk flexion and extension. Crewmembers also had pre- and post-fitness assessments for maximum pushups, situps, and pullups performed in 2 min, maximum fingertip distance during a sit and reach exercise, and maximum weight lifted while performing smith bench presses and leg presses. No logs of in-flight nutrition were available in this subset.

Shapiro-Wilk tests were performed on all exercise frequencies and functional fitness assessments to evaluate for normally distributed results. Functional fitness parameters with normal distributions were individually linearly regressed against lumbopelvic muscle changes to assess for any functional changes as a result of radiologically observed findings. Lumbopelvic muscle changes with normal distributions were individually linearly regressed against CEVIS (min/day), TVIS (min/day), and iRED (uses/day) to evaluate for significant changes resulting from crewmembers’ choice of in-flight fitness routine. These values were determined by taking the total minutes spent using CEVIS or TVIS and total flight uses of iRED and normalizing by the mission duration in days.

## Results

**Figure 2:**
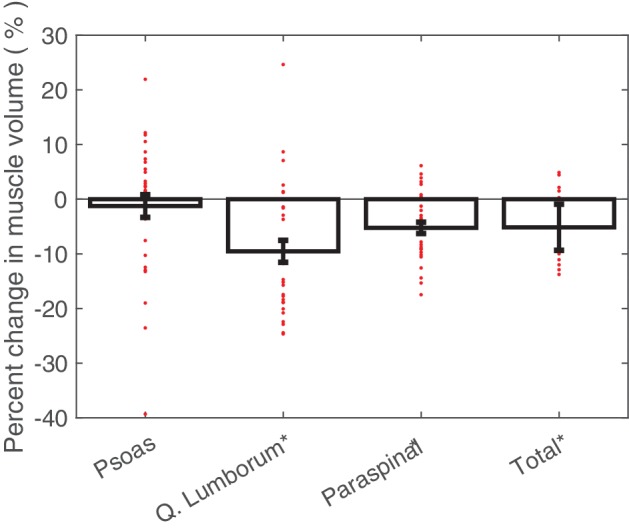
Average percent changes (with standard error bars) in crewmember psoas, quadratus lumborum, paraspinal, and total lumbopelvic muscle volumes, with individual crewmember data points overlaid. Determined from pre- and post-flight manually segmented CT scans of the L2 region. *p < 0.05.

The psoas, quadratus lumborum, and paraspinal muscle volumes were grouped into a single mask to quantify the total change in lumbopelvic muscle volume (Table 1). Fourteen of the 16 (88%) crewmembers showed a decrease in total lumbopelvic muscle volume following spaceflight, ranging from 2.4 to 10.5%. One crewmember showed a 4.7% increase in total lumbopelvic muscle volume following spaceflight. One crewmember showed no change in total lumbopelvic muscle volume from baseline. The total lumbopelvic musculature showed an average 5.1% (4.2% SE; *p* < 0.001) decrease following spaceflight (Figure 2).

**Table 1:** Changes in lumbar musculature along the L2 vertebrae for each crewmember.

Subject	Change in muscle volume (cm^3^)	Percent change in muscle
1	14.7	4.7
2	−30.4	−9.3
3	−4.5	−2.5
4	−30.2	−10.5
5	−12.1	−3.9
6	−12.8	−5.6
7	−32.9	−11
8	−20.5	−6.2
9	0.5	0.2
10	−22.3	−7.3
11	−12.8	−4.2
12	−23.1	−8.6
13	−28.6	−8.9
14	−2.5	−1.3
15	−16.1	−5.4
16	−6.6	−2.4
Mean (SE)	−15.0 (13.2)	−5.1 (4.2)

Post-flight decreases in individual muscle volumes were observed in the crewmembers (Figure 2). Significant decreases in quadratus lumborum muscle volume (mean: 9.5%; 2.0% SE; *p* < 0.0001) and paraspinal muscle volume (mean: 5.3%; 1.0% SE; *p* < 0.0001) were observed. Crewmembers did not experience statistically significant changes in psoas muscle volume.

**Figure 3:**
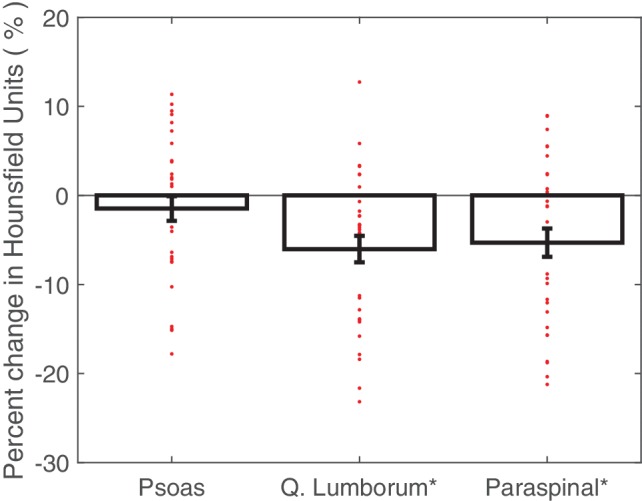
Average percent changes (with standard error bars) in crewmember psoas, quadratus lumborum, and paraspinal muscle attenuation, with individual crewmember data points overlaid. Determined from pre- and post-flight manually segmented CT scans of the L2 region. *p < 0.05.

**Figure 4:**
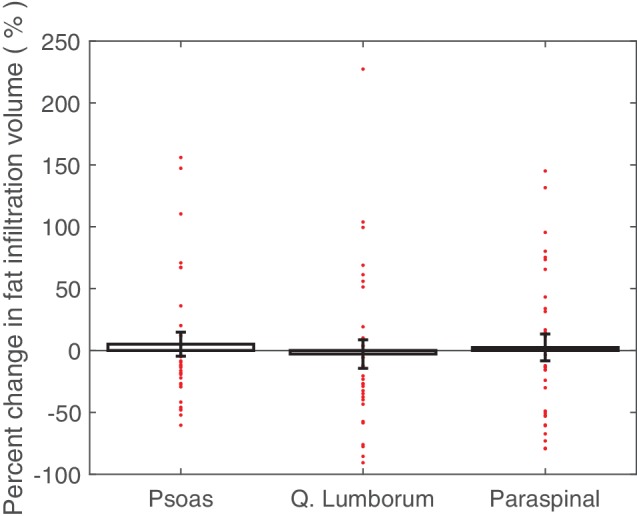
Average percent changes (with standard error bars) in crewmember psoas, quadratus lumborum, and paraspinal muscle fat infiltration mask volume, with individual crewmember data points overlaid. Determined from pre- and post-flight manually segmented CT scans of the L2 region.

The individual muscle groups were assessed to determine the overall change in muscle attenuation (measured in HU; Figure 3). The quadratus lumborum muscle demonstrated a significant HU decrease in 6.0% (1.5% SE, *p* < 0.001). The paraspinal muscles also demonstrated a significant HU decrease in 5.3% (1.6% SE, *p* < 0.01). The psoas muscles showed no statistically significant changes in HU. The individual muscle groups were assessed to determine the overall change in IMAT among the crewmembers. None of the muscles showed significant changes in fat infiltration volumes and both the psoas and quadratus lumborum fat infiltration volumes were non-normally distributed with outliers experiencing substantial increases in fat infiltration volume (Figure 4).

In the subset of individuals with functional fitness assessment test results available, no significant correlations between changes in lumbopelvic muscle volumes or attenuation values were observed with linear regression. However, there were some significant correlations found with lumbopelvic muscle changes when regressed against in-flight iRED and CEVIS use, but not TVIS use.

**Figure 5:**
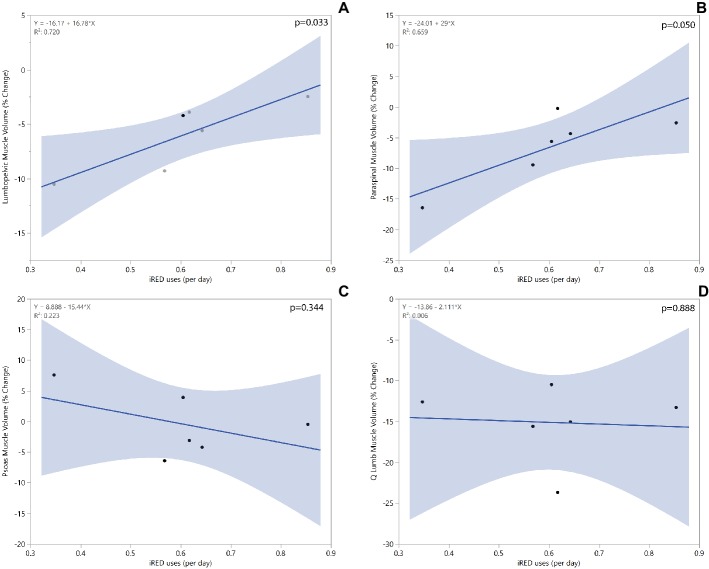
Linear regression models for total lumbopelvic **(A)**, paraspinal **(B)**, psoas **(C)**, and quadratus lumborum **(D)** muscle volume change based on average number of iRED workouts/day while on ISS mission.

A significant regression equation to predict total lumbopelvic muscle volume percent change based on the average number of daily iRED workouts while on ISS missions was found (*R*^2^ = 0.72, *p* < 0.05). Crewmembers were shown to have a 2.4% improvement in lumbopelvic muscle volume retention for each additional weekly iRED workout performed. Crewmembers who used iRED twice a week were found to have about an 11% decrease in lumbopelvic volume, whereas those who used iRED six times a week were found to have about a 2% decrease in lumbopelvic volume. Similar positive correlations based on iRED exercise frequency were found for paraspinal muscle volume but not for quadratus lumborum or psoas muscle volumes (Figure 5). No trends in muscle attenuation were seen with regard to iRED workout frequency.

**Figure 6:**
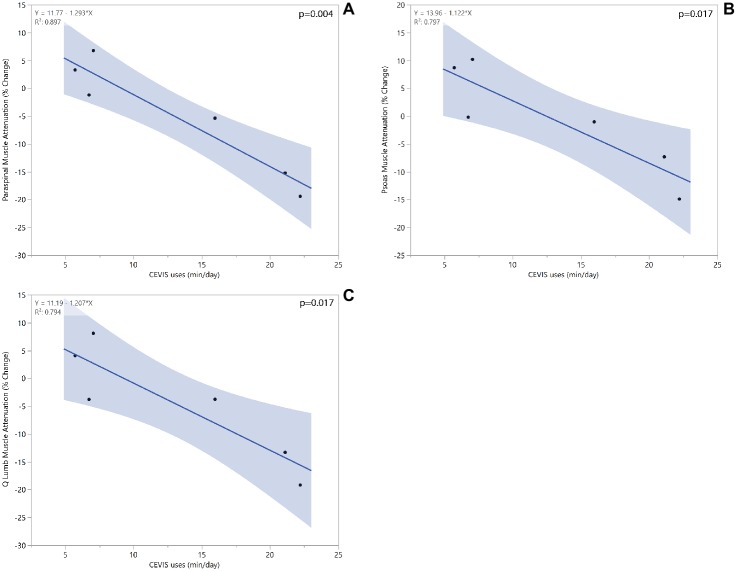
Linear regression models for paraspinal **(A)**, psoas **(B)**, and quadratus lumborum **(C)** muscle attenuation change based on average minutes spent of CEVIS per day while on ISS mission.

A significant regression equation to predict paraspinal muscle attenuation percent change based on the average time spent using CEVIS while on ISS missions was found (*R*^2^ = 0.90, *p* < 0.004). Crewmembers were shown to have a 1.3% decrease in paraspinal muscle attenuation for each daily minute spent using CEVIS. Crewmembers who used CEVIS for 5 min/day were found to have about a 5% increase in paraspinal muscle attenuation, whereas those who used CEVIS for 20 min/day were found to have about a 14% decrease in paraspinal muscle attenuation. Similar negative correlations based on CEVIS exercise duration were found for percent changes in muscle attenuation of the quadratus lumborum and psoas muscles (Figure 6). No trends in muscle volumes were seen with regard to CEVIS workout frequency.

## Discussion

The results of this study support the consensus that long-duration spaceflight is detrimental to the overall lumbopelvic muscle volume in astronauts. The study shows the degree to which different lumbopelvic muscles are affected after the implementation of iRED. It is noteworthy that these iRED-era missions had less severe muscle volume degradation than seen in prior studies (LeBlanc et al., 2000), and that the declines in paraspinal musculature and lack of changes to the psoas are in agreement with results from bed-rest studies (Cao et al., 2005). It is also noteworthy that linear regressions found iRED workouts preserved lumbopelvic muscle volume. Although the paraspinal muscles are still experiencing degradation in space, the volume of muscle degradation has been effectively halved. The psoas muscle is no longer experiencing significant levels of degradation. This may be explained by its function as a hip flexor compared to the paraspinal and quadratus lumborum muscles, which function as posture stabilizers. This discrepancy in degradation of hip flexors and posture stabilizers may indicate that workout equipment is not adequately targeting the muscles responsible for stabilizing the lumbar spine. Loss in these muscles may leave astronauts more susceptible to lumbar injury after spaceflight.

In addition to the volumetric changes exhibited in the muscles responsible for postural stability, the results demonstrate a potential increase in lipid deposition in these muscles. Both the paraspinal and quadratus lumborum muscles exhibited a significant decrease in muscle attenuation. CT muscle attenuation is affected by IMAT. Previous studies have shown that a 1 HU decrease in muscle attenuation corresponds to a 0.01 g ml^−1^ increase in muscle lipid concentration (Goodpaster et al., 2000). This means that approximately 3 HU decrease seen in the quadratus lumborum muscles corresponds to more than 0.03 g ml^−1^ increase in their lipid concentration as a result of prolonged microgravity. A linear regression found that crewmembers spending more time on CEVIS had higher decreases in muscle attenuation. This may indicate that CEVIS is not activating the lumbopelvic musculature in ways to prevent lipid deposition in the muscle fibers. However, it is also possible that this attenuation from CEVIS use is due to a tradeoff in which crewmembers spending more time on CEVIS have less available time to use other in-flight exercise equipment. Studies have found a direct relationship between muscle attenuation and muscle strength even when controlling for cross-sectional area (Goodpaster et al., 2001; Visser et al., 2002). Similarly, many studies have shown an increase in skeletal muscle lipid deposition with age (Ryan and Nicklas, 1999) and with muscle-wasting diseases (Jones et al., 1983; Liu et al., 1993).

The decreases in both muscle volume and quality found in this study may contribute to a decrease strength in lumbopelvic musculature that places crewmembers at risk for injury following spaceflight. Spinal injuries and pain are one of the most common post-flight injuries (Ramachandran et al., 2018). Longitudinal studies of astronauts and matched controls have found a 4.3 times increased incidence of herniated nucleus pulposus in the astronaut population, occurring in 44 of 321 (13.7%) of astronauts (Johnston et al., 2010). In the first-year post-flight, the risk of herniated cervical nucleus pulposus is 35.9 times higher in astronauts than controls (Johnston et al., 2010). Recent studies have found an association between spinal muscle atrophy and decreased lumbar lordosis that can lead to an increased risk of herniated nucleus pulposus (Bailey et al., 2018). These findings further underscore the potential consequences of spinal muscle atrophy quantified in this study, as the decreased strength of lumbar musculature may increase the risk of herniated nucleus pulposus. It is important to highlight the lack of association between the lumbopelvic muscle changes in this study and the crewmembers’ trunk extension peak torque values. It is very likely that with a sample size of 6 individuals, associations that may exist between lumbopelvic muscle changes and functional fitness assessments were not adequately powered.

In-flight countermeasures are of great importance in preventing post-flight injuries (Sayson et al., 2013). However, iRED and its replacement (aRED) lack exercise protocols that mimic spine loading on Earth (Sayson et al., 2013), which may explain why spinal pain has yet to be eliminated with the use of such equipment (Laughlin et al., 2016). NASA’s Astronaut Strength, Conditioning, and Rehabilitation (ASCR) team is continually focused on improving both in-flight exercise regimens as well as post-flight rehabilitation to prevent spinal injuries. While the results of this study demonstrate improvement to the lumbar musculature during the iRED-era, the lumbar musculature was still degrading in comparison to pre-flight values. These declines highlight the continued importance of the ASCR team to aid in the maintenance of muscle health in order to offset in-flight deconditioning and promote a shorter re-conditioning period (LeBlanc et al., 2002, 2013; Zhang et al., 2011; Smith et al., 2012; Lloyd et al., 2015; MacNabb et al., 2016; Lang et al., 2017; Ramachandran et al., 2018). Future studies should look at the effects of the newer aRED on lumbopelvic musculature to determine its efficacy over the retired iRED.

Study limitations include a small sample size (*n* = 16 for muscle changes and *n* = 6 for fitness data). However, there were only 19 crewmembers on ISS missions during the data collection period. Furthermore, the sample included mostly men, with only one female participant. However, there were only two female ISS crewmembers during the data collection period. Another limitation to the scans was the small region of interest, which only spanned the L1–L2 vertebrae. The physiologic changes to the entire muscle were characterized by the changes experienced at the level of the L2 vertebral body. However, the paraspinal muscles run the entire length of the spine, the psoas runs the entire length of the lumbar spine as well as the entire pelvic region, and the quadratus lumborum runs the entire length of the lumbar spine. Previous literature has shown that measurements taken at the belly (site of maximal cross-sectional area) of a muscle can be used to predict overall muscle volume (Albracht et al., 2008). However, the L1–L2 region is closer to the origin/insertion locations of these muscles and may not entirely capture the significance of changes in these muscles.

## Conclusions

Using pre- and post-flight CT scan analysis, we quantified changes in lumbar musculature in crewmembers of long-duration space missions during the iRED era. Through countermeasures including resistive exercise training (e.g., iRED), crewmembers are effectively combating structural deterioration of the lumbopelvic muscles. While these measures appear to have eliminated degradation in the psoas muscles, there are still deleterious effects in the quadratus lumborum and paraspinal muscles. This may leave crewmembers susceptible to back injuries on re-entry and in the months following spaceflight. Future studies should focus on targeting muscles responsible for postural stability in microgravity to prevent their degradation while on missions.

## Ethics Statement

This study was carried out in accordance with the recommendations of the Institutional Review Boards at the Wake Forest School of Medicine and the National Aeronautics and Space Administration (NASA). All subjects gave written informed consent in accordance with the Declaration of Helsinki. The protocol was approved by the Wake Forest School of Medicine and the NASA Institutional Review Boards.

## Author Contributions

LL and AW designed the research. KM, KG, and AM performed the data collection, data analysis, and statistical analysis. LL and AW contributed to data interpretation. KM, KG, LL, and AW wrote and revised the manuscript. AW had primary responsibility for final content. All authors listed have made a substantial, direct, and intellectual contribution to the work and approved it for publication.

### Conflict of Interest Statement

The authors declare that the research was conducted in the absence of any commercial or financial relationships that could be construed as a potential conflict of interest.
